# Protein composition and bread volume of German common wheat landraces grown under organic conditions

**DOI:** 10.1016/j.crfs.2024.100871

**Published:** 2024-10-01

**Authors:** Nora Jahn, Ulla Konradl, Klaus Fleissner, Sabrina Geisslitz, Katharina A. Scherf

**Affiliations:** a Department of Bioactive and Functional Food Chemistry, Institute of Applied Biosciences, Karlsruhe Institute of Technology (KIT), Adenauerring 20 a, 76131, Karlsruhe, Germany; b Bavarian State Research Center for Agriculture (LfL), Kleeberg 14, 94099, Ruhstorf a.d.Rott, Germany; c Leibniz Institute for Food Systems Biology at the Technical University of Munich, 85354, Freising, Germany; d Technical University of Munich, TUM School of Life Sciences, Food Biopolymer Systems, Freising, Germany

**Keywords:** Ancient, Biodiversity, Bread making, Gluten, Modern varieties, Old varieties

## Abstract

Landraces are genetically heterogeneous plant populations that are regionally particularly well adapted to the natural and cultural agricultural environment. Their genetic memory originates from pre-industrial agriculture and food production with consequences for their agronomic and processing performance. Since wheat-related disorders have increased in the population, breeding might have resulted in changes in the protein composition. The aim of this study was to investigate the protein composition and baking quality of 14 German common wheat landraces. Six modern varieties served as a control group. The protein composition was determined using modified Osborne fractionation and reversed-phase high-performance liquid chromatography. In addition, the water absorption and bread volume were determined.

The crude protein content, proportions of albumins and globulins, water absorption and bread volume did not differ between modern varieties and landraces. The proportion of gliadins was higher in landraces (64.0%) compared to modern varieties (57.6%), whereas the proportion of glutenins was lower in landraces (17.4%) than in modern varieties (22.0%). The same observation was made for the respective gluten protein types except the proportion of ω1,2-gliadins, where there was no difference between the two groups. This resulted in a significantly higher ratio of gliadins to glutenins of 4.3 in landraces compared to 2.8 in the modern varieties, but no difference in the total gluten proportion. Taken together, there was no clear distinction between landraces and modern varieties. However, a few landraces such as *Roter Sächsischer Landweizen* showed similar characteristics to modern varieties and are therefore interesting for further investigations.

## Highlights

• Protein and gluten content of wheat landraces and modern varieties did not differ.
• Protein composition was slightly different.
• Gliadin/glutenin ratio was higher in landraces.
• Water absorption and bread volumes were similar.
• Common wheat landraces can be a regional alternative to modern varieties.

## Graphical abstract

**Figure:**
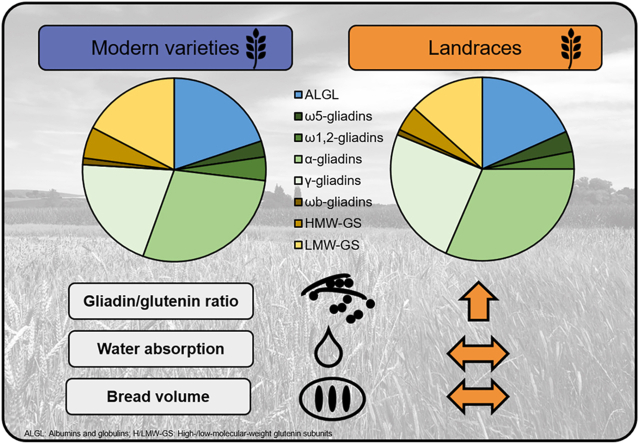

## Abbreviations

ALGL: albumins and globulins
ANOVA: analysis of variance
ATI: amylase/trypsin-inhibitor
CD: celiac disease
GLIA/GLUT: gliadin/glutenin ratio
HMW-GS: high-molecular-weight glutenin subunits
LMW-GS: low-molecular-weight glutenin subunits
NCWS: non-celiac wheat sensitivity
PCA: principal component analysis
PWG: Prolamin Working Group
RP-HPLC: reversed-phase high-performance liquid chromatography
WA: wheat allergy

## 1 Introduction

Ancient grain varieties such as landraces have recently gained increasing attention, because certain consumers consider them as natural, healthy and environmentally friendly (Teuber et al., 2016) and associate products made thereof with a better tolerability (Seidita et al., 2022). There are several different definitions for the term landrace, but with certain similarities. The first attribute is the adaptation to local agro-environmental conditions. Therefore, landraces are often closely associated with one specific geographical location. Another attribute is the involvement in alternative farming systems such as organic agriculture. Furthermore, landraces have largely developed as a result of time and natural selection, hence providing a large genetic diversity (Villa et al., 2007). This raises the question, if the selection criteria during breeding towards higher yields and increased plant resistance may have resulted in changes in the protein composition and thus increased the immunoreactive potential in modern varieties (Scherf, 2019).

This question is of great importance because the prevalence of wheat-related disorders has increased in the population. These are differentiated according to their pathomechanism into celiac disease, wheat allergy (WA) and non-celiac wheat sensitivity (NCWS) (Sapone et al., 2012).

Cereal proteins can be classified into Osborne fractions according to their solubility. Amylase/trypsin-inhibitors (ATIs), along with other enzymes and enzyme-inhibitors belong to the water- and salt-soluble albumins and globulins (ALGL). Gluten proteins are storage proteins and can be further divided into gliadins and glutenins. Gliadins are soluble in aqueous alcohols and mainly occur as monomers. A complete extraction of polymerized glutenins is performed at increased temperatures by solvents containing a mixture of aqueous alcohols, reducing agents and disaggregating compounds (Wieser et al., 2014). Gluten peptides are triggers for CD and WA (Kissing Kucek et al., 2015).

Gluten proteins can be further classified with reversed-phase high-performance liquid chromatography (RP-HPLC) (Schalk et al., 2017; Wieser et al., 1998). Gliadins contain ω5-, ω1,2-, α- and γ-gliadins. Glutenins are divided into high- (HMW-GS) and low-molecular-weight glutenin subunits (LMW-GS) and ωb-gliadins. The latter appear in the glutenin fraction, because they are modified by substitution of a single amino acid residue for a cysteine residue that is linked to other gluten proteins by a disulfide bond (Wieser et al., 2022).

All fractions contain immunoreactive proteins, with most of the proteins also comprising CD-active peptides (Lexhaller et al., 2019). The most reactive gluten proteins for triggering CD and baker's asthma (the most common form of WA) are mostly α- and ω-gliadins, but also γ-gliadins, HMW-GS and LMW-GS to a lesser extent. For wheat-dependent exercise-induced anaphylaxis (WDEIA), the ω5-gliadins are the main triggers (Kissing Kucek et al., 2015).

When mixed with water, gluten forms a viscoelastic dough. An optimal ratio between gliadins and glutenins (GLIA/GLUT) enables ideal gas holding properties (Scherf, 2019). High contents of glutenins and HMW-GS and low GLIA/GLUT are correlated to the bread volume and can therefore be used as predictors of baking quality (Geisslitz et al., 2018). Despite lower yields compared to modern varieties (Denčić et al., 2000), landraces are already in use for the production of breads. An example of a success story for the use of landraces for organic food specialties is that of the *Laufener Landweizen*, which was rediscovered about twenty years ago and is now very popular in the Salzburg - Berchtesgadener Land - Traunstein region in Austria and south-eastern Germany (Adelmann et al., 2018).

Currently, there are few studies comparing landraces with modern wheat varieties grown under organic conditions. Skendi et al. (2023) analyzed Greek landraces of different wheat varieties, including two commercial flours for comparison. One result of this study was that high-quality breads can be produced by using a traditional bread-making procedure. Sciacca et al. (2018) evaluated twelve Sicilian durum wheat landraces and three modern varieties as a control group regarding their macro- and micronutrients and found higher concentrations of N, P, K, Fe, Zn, Mn and Sr in the landraces. In the study of Migliorini et al. (2016) five bread samples baked with landraces were rated against a bread made out of a modern variety in terms of appearance, smell and taste. On average, the breads produced with the old varieties were preferred over the control bread. Some studies focused on the effect of breeding on changes in gluten protein composition of common wheat varieties (Pronin et al., 2020; Call et al., 2020; Ozuna and Barro, 2018), but with no focus on landraces.

Comprehensive studies regarding the protein composition on a well-defined sample assortment of landraces and modern varieties are completely lacking. Therefore, this study aimed to investigate the protein composition and baking quality of 14 common wheat landraces and a control group of six modern varieties cultivated under organic conditions in three consecutive years. This will be an important step toward the overall objective to meet consumer demands for regional and healthy organic specialties.

## 2 Materials and methods

### 2.1 Materials

Fourteen German landraces and six modern varieties of common wheat (*Triticum aestivum* L.) were cultivated at the Bavarian State Research Center for Agriculture (Ruhstorf an der Rott, Germany) and harvested in 2021, 2022 and 2023 (Table 1). One variety was only available in two harvest years (WIW). The cultivation was carried out under organic growing conditions without fertilization. The experimental layout was a randomized complete block design with three replications, which were harvested and milled together. The kernels were milled with a Quadrumat Junior (Brabender, Duisburg, Germany) to obtain type 550 flours (ash content of 0.51%–0.63% based on dry matter) according to the German flour classification system.

**Table 1:** Overview of common wheat varieties. Asterisks indicate a modern variety.

Species	Variety	Abbreviation	Harvest years
*T. aestivum* ssp. *aestivum*	Ackermanns Bayernkönig	ABK	2021, 2022, 2023
	Alpiner begrannter Land	ABL	2021, 2022, 2023
	Altbanater	ALT	2021, 2022, 2023
	Berchtesgadener Vogel	BEV	2021, 2022, 2023
	Eglfinger Hohenstaufen	EGH	2021, 2022, 2023
	Freisinger Landweizen	FLW	2021, 2022, 2023
	Niederbayerischer Braun	NBR	2021, 2022, 2023
	Nördlinger Roter	NOR	2021, 2022, 2023
	Roter Sächsischer Landweizen	RSL	2021, 2022, 2023
	Schwäbischer Dickkopf Landweizen	SDL	2021, 2022, 2023
	Unterfränkischer Land	UNL	2021, 2022, 2023
	Wahrberger Ruf	WAR	2021, 2022, 2023
	Wetterauer Fuchs	WEF	2021, 2022, 2023
	Boss∗	BOS	2021, 2022, 2023
	Elixer∗	ELX	2021, 2022, 2023
	KWS Sharki∗	KWS	2021, 2022, 2023
	RGT Reform∗	RGT	2021, 2022, 2023
	Wendelin∗	WEN	2021, 2022, 2023
	Wiwa∗	WIW	2022, 2023
*T. aestivum* ssp. *compactum*	Weihenstephan Igelweizen	WEI	2021, 2022, 2023

### 2.2 Crude protein content

The nitrogen content of the flours was determined in triplicate by the Dumas combustion method (ICC-Standard 167, 2000) using a Dumatherm Nitrogen analyzer (Gerhardt Instruments, Königswinter, Germany). Crude protein content was calculated by using a factor of 5.71.

### 2.3 Osborne fractionation and RP-HPLC analysis

The extraction of wheat proteins was performed according to the modified Osborne fractionation (Wieser et al., 1998). ALGL, gliadins and glutenins were extracted in triplicates stepwise from the flour (100 mg) with the following solutions. A: 0.4 mol/L of NaCl+ 0.067 mol/L Na_2_HPO_4_/KH_2_PO_4_ (pH 7.6), B: 60% (v/v) ethanol, C: 50% (v/v) 1-propanol + 2 mol/L of urea +0.05 mol/L of Tris-HCl (pH 7.5) + 1% (w/v) dithiothreitol. First, ALGL were extracted twice using 1 mL of solution A each, followed by the extraction of gliadins three times using 0.5 mL of solution B. Glutenins were extracted twice using 1 mL of buffer C. Each extraction step was started by 2 min of vortex mixing, followed by magnetic stirring for 10 min at 22 °C (ALGL, gliadins) and 30 min at 60 °C (glutenins), respectively. Then, a centrifugation step was carried out (30 min, 25 °C, 3550 rcf). The extracts were diluted to 2 mL with the respective extraction solution and filtered through a 0.45 μm syringe filter with a regenerated cellulose membrane (WICOM, Heppenheim, Germany). The filtered extract was used for analysis by RP-HPLC, using the parameters reported in Xhaferaj et al. (2023). External calibration was performed using Prolamin Working Group (PWG)-gliadin (distributed by Arbeitsgemeinschaft Getreideforschung e.V., Detmold, Germany), dissolved in 60% ethanol, ultrasonicated and filtered (2.5 mg/mL) (van Eckert et al., 2006). The injection volumes were adjusted so that the resulting areas were in the middle range of the calibration. Typical injection volumes were: ALGL, 20–30 μL, gliadins 5–10 μL, glutenins 15–30 μL. The content of ALGL, gliadins and glutenins was calculated using the corresponding total peak area. The gluten protein types ω5-gliadins, ω1,2-gliadins, α-gliadins and γ-gliadins were quantitated based on their percentage of the total peak area of gliadins. HMW-GS, LMW-GS and ωb-gliadins were quantitated based on their percentage of the total peak area of glutenins.

### 2.4 Baking tests

For the baking tests, 200 g of each flour were mixed with water as determined in the farinograph (Brabender), 6% yeast (Wieninger, Teisendorf, Germany), 2% sugar, 2% fat (Goldbiskin Meistermarken, CSM Deutschland, Bremen, Germany), 0.002% ascorbic acid and the required amount of malt flour to obtain a falling number of 250 s. Doughs were prepared at a temperature of 26 °C and rested for 30 min, 5 min of which were for processing. This was followed by 30 min of piece proofing (32 °C and 80% relative humidity). Breads were baked for 30 min at 230 °C. Bread volume was determined by measurement of the volume of rapeseed that is displaced by the bread.

### 2.5 Statistical analysis

Mean values and standard deviations of triplicates were calculated with Microsoft Office Excel 2016 (Microsoft Corporation, Seattle, WA, USA). Statistical evaluation using Pearson correlations, one-way analysis of variance (ANOVA) with Tukey's Test (p < 0.05), two-way ANOVA with the factors variety and year as well as principal component analysis (PCA) were performed using OriginPro 2023 (OriginLab, Northampton, MA, USA). Pearson correlation coefficients (r) were defined as follows: ±0.54<r≤±0.67: weak correlation; ±0.67<r≤±0.78: medium correlation; ±0.78<r ≤±1.00: strong correlation (Thanhaeuser et al., 2014).

## 3 Results

### 3.1 Crude protein content

**Fig. 1:**
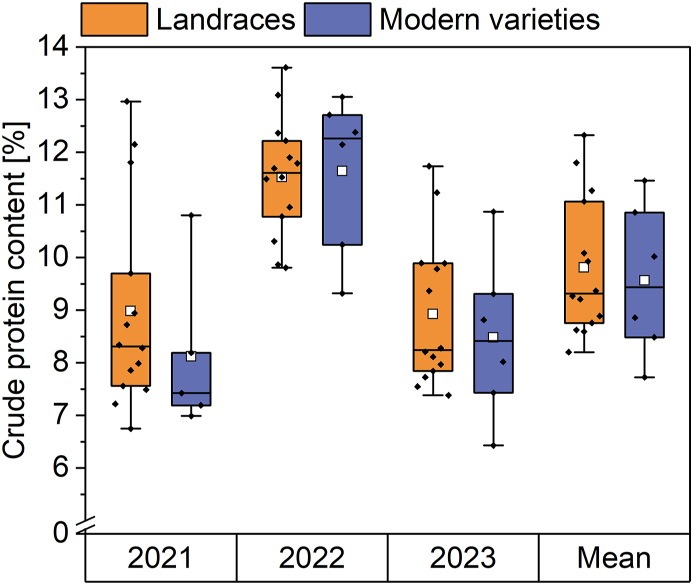
Crude protein content of landraces (n = 14) and modern varieties (n = 5–6). Boxplots display the interquartile range (box), mean value (white square), median (horizontal line) and minima and maxima (whiskers). Single cultivars are displayed within the boxplots (black diamond).

The crude protein content of landraces and modern varieties was analyzed in flours from the harvest years 2021, 2022 and 2023 and a mean was calculated over all three years for each sample (Fig. 1., Table S1). Considering all three years separately, the protein content of the landraces ranged from 6.7% (WAR in 2021) to 13.6% (SDL in 2022), while that of the modern varieties ranged from 6.4% (ELX in 2023) to 13.1% (WEN in 2022). This gives a first indication that the two groups do not differ. In 2021, the mean of the landraces was 9.0%, while that of the modern varieties was a bit lower (8.1%). The smallest difference between the mean of both groups occurred in 2022 with values of 11.5% for the landraces and 11.6% for the modern varieties. Similar to 2021, in 2023, the mean of the landraces was slightly higher (8.9%) compared to the modern varieties (8.5%).

All in all, there were only minor differences between the two groups in all three years separately. This was confirmed when looking at the mean over three years, which resulted in a mean crude protein of 9.8% for the landraces and 9.6% for the modern varieties. Considering the mean values of all three years, the landrace FLW had the highest protein content (12.3%), while the modern variety ELX had the lowest (7.7%). Looking at the different years in general, the harvest years 2021 and 2023 resulted in similar crude protein contents for both the landraces and the modern varieties. The year 2022 stands out because the crude protein content was the highest for almost all varieties. The only exception was the landrace WEI, which had the highest protein content in 2021 (13.0%). As no difference in crude protein content was found between landraces and modern varieties, the protein composition was examined more closely.

### 3.2 Quantitation of ALGL, gliadins and glutenins

The proportions of ALGL, gliadins and glutenins were calculated relative to the total protein content (sum of ALGL, gliadins and glutenins) in order to eliminate the variations in protein content over the three years. Furthermore, the values of the three harvest years were averaged to determine differences between the varieties regardless of environmental factors.

**Fig. 2:**
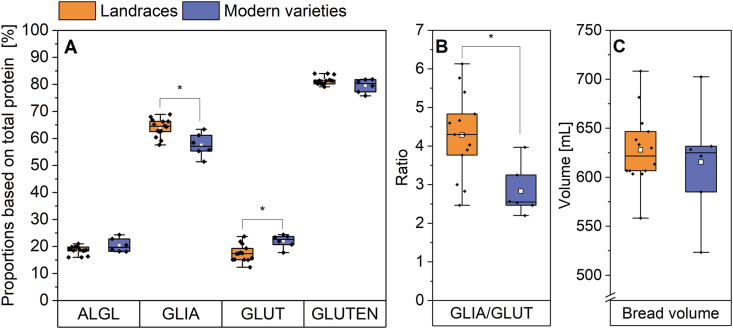
Protein composition and bread volume of landraces (n = 14) and modern varieties (n = 6) as mean of all three harvest years. The results are displayed as in Fig. 1. Asterisks indicate significant differences between landraces and modern varieties (one-way ANOVA, Tukey's test at p < 0.05). A: Relative proportions of albumins and globulins (ALGL), gliadins (GLIA), glutenins (GLUT) and gluten based on total protein (sum of ALGL, GLIA and GLUT); B: Gliadin/glutenin ratio (GLIA/GLUT); C: Bread volume.

The proportion of ALGL was slightly higher within the modern varieties (20.5%) than in the landraces (18.6%), but there was no significant difference (Fig. 2A). The landraces FLW and SDL had the lowest ALGL proportion (16.0%, each), while three of the four highest ALGL proportions were present in the modern varieties RGT (24.3%), BOS (22.8%) and ELX (20.5%) (Table S2).

The proportion of gliadins ranged from 51.4% (RGT) to 68.9% (FLW). In general, the gliadin proportion of landraces was significantly higher (64.0%) compared to modern varieties (57.6%) (Fig. 2A). In contrast, the proportions of glutenins were significantly lower within the landraces (17.4%) than within the modern varieties (22.0%). The modern variety RGT (24.4%) had the highest glutenin proportion and it also had the highest ALGL proportion and the lowest gliadin proportion. Since the mean gliadin proportion was higher and the glutenin proportion lower in landraces, the total gluten content was similar in landraces (81.4%) and modern varieties (79.6%).

Almost the same results were obtained regarding the absolute content (Fig. S1, Table S3). With landraces having 19.3 mg/g and modern varieties 20.0 mg/g of flour, there was also no difference in the ALGL content. The gliadin content was higher in the group of landraces (67.9 mg/g) than in the group of modern varieties (58.4 mg/g) as well. The glutenin content was slightly higher in the group of modern varieties (22.0 mg/g) compared to the landraces (18.7 mg/g). As consequence, the absolute gluten content was slightly higher in the landraces (86.6 mg/g) compared to the modern varieties (80.4 mg/g). In contrast to the relative proportions, there were no significant differences.

The mean GLIA/GLUT of the samples averaged over all three harvest years ranged from 2.2 (RGT) to 6.1 (WAR) (Fig. 2B). The mean of the landraces (4.3) was significantly higher than that of the modern varieties (2.8). In all three years, all the modern varieties had GLIA/GLUT between 1.8 and 5.6, while the range of all samples of the landraces was between 2.0 and 10.9, having values up to two times higher than the mean of modern varieties (Table S2). A few landraces featured low GLIA/GLUT in all three years, such as WEF (2.1, 3.2, 3.2) and EGH (2.4, 2.3, 2.7). With a mean value of 6.0 (landraces) and 3.4 (modern varieties), the year 2023 showed the highest ratio of all three years (3.3 and 2.4 for 2021 and 3.5 and 2.6 for 2022, respectively) (Table S4).

### 3.3 Quantitation of gluten protein types

**Fig. 3:**
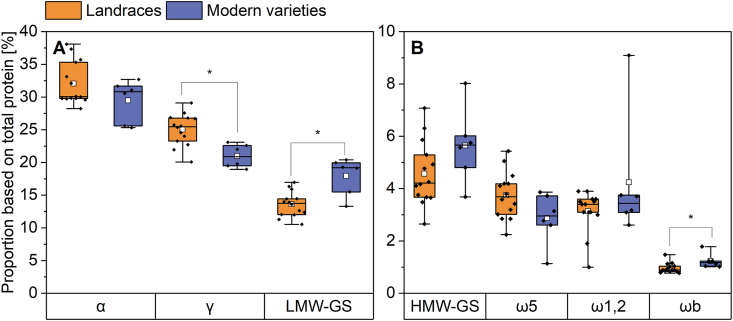
Gluten composition of landraces (n = 14) and modern varieties (n = 6) as mean of all three harvest years. The results are displayed as in Fig. 1. Asterisks indicate significant differences between landraces and modern varieties (one-way ANOVA, Tukey's test at p < 0.05). A: Relative proportions of α-gliadins (α), γ-gliadins (γ) and low-molecular-weight glutenin subunits (LMW-GS); B: Relative proportions of high-molecular-weight glutenin subunits (HMW-GS), ω5-gliadins (ω5), ω1,2-gliadins (ω1,2) and ωb-gliadins (ωb).

Gluten proteins were further divided into ω5-, ωb-, ω1,2-, α- and γ-gliadins as well as HMW-GS and LMW-GS. The mean proportions of gluten protein types are shown in Fig. 3. Individual values can be found in Table S5. For both groups, α-gliadins had the highest proportions of total protein (28.2–38.1% for landraces and 25.3–32.7% for modern varieties), followed by γ-gliadins (20.1–29.1% and 19.0–23.1%, respectively). LMW-GS ranged between 10.5% and 17.0% for landraces and between 13.3% and 20.4% for the modern varieties. HMW-GS values were 2.7–7.1% and 3.7–8.0%, respectively. For the landraces, ω5-gliadins had a higher proportion (2.2–5.4 %) than the ω1,2-gliadins (1.0–3.9%). For the modern varieties, it was the other way around as the proportion of ω1,2-gliadins (2.6–9.1 %) was higher than the one of the ω5-gliadins (1.1–3.9%). The ωb-gliadins had the smallest proportion of gluten protein types with values between 0.8% and 1.5% (landraces) as well as between 1.0% and 1.8% (modern varieties).

The mean value of the α-gliadins was slightly higher in the group of landraces (32.0%) than in the one of modern varieties (29.5%). The landrace WAR had the highest α-gliadin proportion with 37.3%, whereas the modern variety BOS had the lowest proportion (25.3%). The same was true for γ- and ω5-gliadins, having mean values of 25.0% and 3.7% for the landraces and 21.0% and 2.9% for the modern varieties, although the difference was only significant for the γ-gliadins. The mean value of ω1,2-gliadins was higher in the modern varieties (4.3%) than in the landraces (3.1%), but only because the sample BOS had a very high proportion of 9.1%. This modern variety BOS had significantly higher values compared to the other modern varieties in all three harvest years (8.2%, 9.5% and 9.6%). Without considering this variety, there was no difference between the two groups over all three years (3.3% and 3.1%, respectively). Taken together, all gliadin types except ω1,2-gliadins were higher in the group of landraces. This is in accordance with the protein composition (chapter 3.2), indicating a higher gliadin proportion for the landraces as well.

All glutenin types were higher in the group of modern varieties, although there was a significant difference only for ωb-gliadins and LMW-GS. This is also in accordance with the protein composition, because the modern varieties had a higher glutenin proportion in general. The ωb-gliadins had a mean value of 1.0% in the group of landraces and of 1.2% in the group of modern varieties. The LMW-GS of landraces and modern varieties had relative proportions of 13.6% and 17.9%, respectively. The mean value of HMW-GS in modern varieties was 5.7% compared to 4.6% in the group of landraces. The modern variety BOS (8.0%) had the highest value, the landrace WAR (2.7%) the lowest. Since the content of glutenins and especially of HMW-GS is correlated with the bread volume (Geisslitz et al., 2018), these results might suggest a better baking quality of the modern varieties. To test this hypothesis, baking tests were performed.

### 3.4 Water absorption and bread volume

**Fig. 4:**
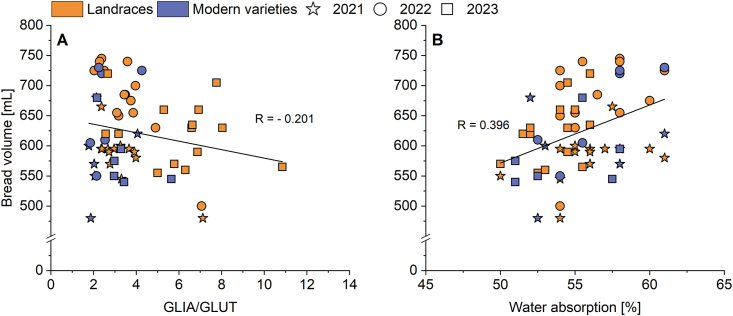
Correlation diagrams of A: Bread volume and gliadin/glutenin ratio (GLIA/GLUT) and B: Bread volume and water absorption of all samples. The different symbols each indicate a different harvest year.

The mean water absorption over three years did not differ between landraces (55.3%) and modern varieties (55.4%). This was also true when looking at the different years separately (Table S4). The mean value of the bread volume was also not significantly different in both groups (Fig. 2C). The breads made of landraces had volumes between 558 mL (WAR) and 708 mL (EGH). The bread volume of modern varieties was between 585 mL (RGT) and 703 mL (WIW). The variety ELX (modern variety) showed values much lower than the mean in all three years individually (2021: 480 mL, 2022: 550 mL, 2023: 540 mL). The opposite was true for the landrace EGH, which had higher values than the mean in all three years (2021: 665 mL, 2022: 740 mL, 2023: 720 mL). No correlation was found (r = −0.201) between GLIA/GLUT and the bread volume of all varieties when looking at all three years individually (Fig. 4A). There was no clear separation between the group of landraces and the group of modern varieties, although the modern varieties tended to cluster at the bottom of the graph, having low GLIA/GLUT (2–4) and bread volumes between around 500 and 625 mL. This figure also shows that a rough distinction between the different harvest years could be made, regardless of whether it was a landrace or a modern variety. Most varieties from 2021 tended to have relatively low GLIA/GLUT (2–4, with exception of 7.1 of FLW) and bread volumes up to 600 mL (some varieties had higher volumes up to 680 mL). Varieties from 2022 also had GLIA/GLUT between 2 and 4 but had higher bread volumes between 600 and 745 mL compared to 2021. Samples from the harvest year 2023 showed the largest range of GLIA/GLUT and bread volume with values between 2.2 and 10.9 and between 540 and 720 mL, respectively. Fig. 4B shows no correlation between the water absorption and the bread volume (r = 0.396). In contrast to Fig. 4A, no cluster formation for the two groups (landraces and modern varieties) was apparent. Nevertheless, minor differences between the three harvest years could be recognized. Water absorption of varieties from 2021 had the highest range (50.0–61.0%), while the varieties of 2022 had a water absorption between 52.5 and 61.0%. The lowest water absorption was found for the harvest year 2023, with most of the varieties having values between 51.0 and 57.5%.

### 3.5 Impact of environmental effects on protein composition

Two-way ANOVA was performed to find out, if the environmental factor (harvest year) or the genetic background (landrace or modern variety) had a more pronounced influence on the protein content and composition (Table S6). For the protein content, only the harvest year had an influence (F = 20.3, p < 0.0001), as the genetic background was insignificant (F = 0.81, p = 0.3708). The influence of the environmental factors on the protein content is further described in the discussion part. For the ALGL proportion, both the year (F = 11.2, p < 0.0001) and the variety (F = 11.9, p = 0.0011) had an equal influence. However, the gliadins were more influenced by genetics (F = 21.8, p < 0.0001) than the environment (F = 8.67, p = 0.0006). The same was true for the glutenins (F = 12.7, p = 0.0008; F = 8.9, p = 0.0005). This is in accordance with the results of the protein composition showing a significant difference between the gliadin and glutenin proportions between the landraces and the modern varieties.

### 3.6 Comparison of landraces and modern varieties by PCA

**Fig. 5:**
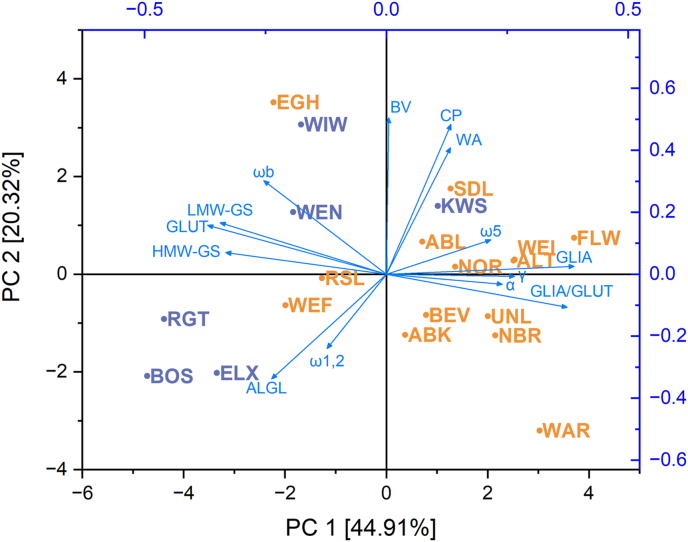
Principal component analysis biplot based on crude protein (CP) content, ratio between gliadins and glutenins (GLIA/GLUT), proportions of albumins and globulins (ALGL), gliadins (GLIA), glutenins (GLUT), α-gliadins (α), γ-gliadins (γ), ω5-gliadins (ω5), ω1,2-gliadins (ω1,2), high- (HMW-GS) and low-molecular-weight glutenin subunits (LMW GS), bread volume (BV) and water absorption (WA) using the mean value of the three harvest years of each cultivar. Abbreviations for the cultivars can be found in Table 1.

The biplot of the component score and variable loadings of the PCA based on crude protein content, GLIA/GLUT and proportions of ALGL, gliadins, glutenins, α-gliadins, γ-gliadins, ω5-gliadins, ω1,2-gliadins, ωb-gliadins, HMW-GS and LMW GS, bread volume and water absorption using the mean value of the three harvest years of each variety is displayed in Fig. 5. Principal component (PC)1 and PC2 made up a total of 65.23% of the total variance. PC1 is mainly composed of the parameters gliadins, glutenins and GLIA/GLUT while bread volume and crude protein mostly contribute to PC2. There was no clear distinction between landraces and modern varieties. However, eleven of the 14 landraces were clustered in the area of the loadings of ω5-, γ- and α-gliadins as well as those of the total gliadin proportion and GLIA/GLUT. Since α- and γ-gliadins made up the largest share of gliadins, it is obvious that the variable loadings all point in the same direction. The varieties WEI and ALT, for example, had almost the same component score, showing that they were similar, especially in terms of the proportions of total gliadins and ω5-gliadins. Also, the landraces BEV/ABK and UNL/NBR were similar to each other, mostly because of the similar GLIA/GLUT (4.0/3.8 and 4.6/4.8) and proportion of ω1,2-gliadins (3.4/3.1 and 3.6/3.4). The landrace WAR stood out because it is located in the right lower corner of the PCA with no other varieties close by. This can be explained by the high GLIA/GLUT (6.1). Except for the variety KWS, which was placed within the group of landraces close to the landrace SDL, all modern varieties were located on the negative side of the axis of PC1. The modern varieties WEN and WIW were clustered in the upper left corner, mostly because of their high proportions of ωb-gliadins and their high bread volume. The three modern varieties RGT, BOS and ELX were all clustered in the lower left corner of the PCA, between the loadings of HMW-GS and ALGL, showing that these samples all had relatively high proportions of ALGL and HMW-GS. The variety BOS stood out due to its high proportion of ω1,2-gliadins. A few landraces were found in between the group of modern varieties. The variety EGH was close to the modern variety WIW, both having relatively high bread volumes (708 and 703 mL, respectively). The landraces RSL und WEF were located between the group of RGT, BOS, ELX and WEN as well as WIW. The three varieties EGH, RSL and WEF also had the lowest GLIA/GLUT within the group of landraces (2.5, 3.0 and 2.8), comparable to those of the modern varieties. Further, the two landraces RSL and WEF had high proportions of HMW-GS (4.9% and 3.8%). All in all, the PCA shows that there are differences between landraces and modern varieties, but that the parameters analyzed are not sufficient to differentiate between them.

## 4 Discussion

The crude protein content of landraces and modern varieties in our study was mostly influenced by environmental factors. Pronin et al. (2020) who analyzed varieties first registered between 1891 and 2010 grown in three years also found that the harvest year had a more significant effect on the crude protein content than the variety. The mean values of landraces and modern varieties in our study did not differ significantly. Prandi et al. (2017) also found no trends for protein content between old (already existing before 1914) and modern (first developed after 1918) *Triticum* varieties. In our study, the protein content was higher in 2022 compared to the other two years. Since the location where the varieties were grown was the same in all three years and there was no fertilization, the environmental factors include mainly climatic factors. Temperatures, precipitation and hours of sunshine have different effects on protein content and composition. Especially when plants do not receive (high levels of) fertilizer, the protein content may be increased by heat, which may also lead to differences in the protein composition (Dupont and Altenbach, 2003). According to Vollmer and Musshoff (2018), the impact of weather conditions on the protein content may vary depending on the month. Higher temperatures in May and July lead to a higher protein content. Since the mean value for May was a lot higher in 2022 (16.0 °C) than the ones in 2021 and 2023 (11.8 and 14.8 °C) (see Table S7) this could be one explanation for the higher protein content in 2022. Higher precipitation in April and June also leads to a higher protein content and high precipitation in July leads to a lower content. Because in 2022 the precipitation in June was higher (103.2 mm) than in the two other years (90.6 and 32.5 mm) and in July those of 2021 and 2023 were a lot higher than in 2022 (118.8 and 101.7 mmm compared to 39.9 mm), this could be another explanation for the higher protein content in 2022. In general, it is noticeable that in 2022 there was low precipitation in February, March and July compared to 2021 and 2023. However, it should be noted that other environmental factors could also have an influence on the protein content.

With mean crude protein values over all three years of 9.8% (landraces) and 9.6% (modern varieties), our values are slightly lower than common wheat varieties from other studies (Geisslitz et al., 2018, 2019; Call et al., 2020), probably because the plants were grown under organic agriculture, so no fertilizer was used. Although we could not see any difference in the protein content of landraces and modern varieties, the cultivation method can have a different influence on both groups. Landraces are often associated with organic agriculture. Their quality traits surpass those of high-yielding modern cultivars in organic farming systems (Villa et al., 2007). They are also more resilient because they are able to grow in marginal areas and regions with high local demands (Zencirci et al., 2021). Therefore, landraces represent a great opportunity for the future to adapt well to specific growing conditions. This is particularly interesting in terms of global warming.

We showed that the mean proportion of ALGL of modern varieties (20.6%) was in the range of common wheat reported by Geisslitz et al. (2018) (20.3–32.0%). That of landraces was slightly lower (18.6%), but there was no significant difference. This is in accordance with Pronin et al. (2020) who found no changes in the ALGL content of varieties first registered between 1891 and 2010.

In our study, there was no difference in the total gluten content (sum of gliadins and glutenins) of landraces (81.4%) and modern varieties (79.6%). This is in contrast to the results of Ozuna and Barro (2018) who observed a significant decrease in the gluten content in domesticated species. Pronin et al. (2020) support our results that there were no changes in total gluten content, but in the gluten composition. Here, the proportions of total gliadins were significantly higher in the group of landraces (64.0%) compared to the modern varieties (57.6%). The types α-, γ- and ω5-gliadins were also higher, although the difference was only significant for γ-gliadins. Call et al. (2020) also investigated the effect of breeding on wheat protein composition. Their study confirmed our findings that old varieties had higher proportions of α- and γ-gliadins. Pronin et al. (2020) reported similar results, showing a decreasing trend for α- and γ-gliadins, but no changes of ω5-and ω1,2-gliadins between 1891 and 2010. A significantly lower α-gliadin proportion in cultivated wheat varieties compared to the wild ancestors of both bread and durum wheat was also found by Ozuna and Barro (2018). We found that contrary to the gliadins, the proportion of glutenins was significantly lower in the group of landraces (17.4%) than in the modern varieties (22.0%). This was also true for the proportions of HMW-GS and LMW-GS, although the difference was only significant for the LMW-GS. Lower proportions of glutenins and glutenin subunits for the old varieties were also confirmed by the study of Pronin et al. (2020). A higher HMW-GS content in modern varieties was also found by Call et al. (2020) and Pronin et al. (2020). This is important because a high HMW-GS content and a low GLIA/GLUT is known to be positively correlated with a high bread volume (Geisslitz et al., 2018). In our study, the higher proportion of gliadins and lower proportion of glutenins in the landraces led to a higher GLIA/GLUT. GLIA/GLUT in landraces (4.3) was significantly higher than that in modern varieties (2.8). The same trend was observed by Call et al. (2020). Typical GLIA/GLUT in modern varieties are between 2.0 and 3.2 (Geisslitz et al., 2018). In our study there was neither a correlation between GLIA/GLUT and bread volume (r = −0.201) nor between HMW-GS and bread volume (r = 0.505). There was no difference between the bread volumes of landraces (628 mL) and modern varieties (615 mL). These results were confirmed by Preiti et al. (2022), although the volumes were generally lower than ours, which is probably due to the different recipe and method of preparation. In their study, landraces had a bread volume of 413 mL, modern varieties had a volume of 418 mL. Water absorption is also very important for the baking quality of wheat. When mixed with water, gluten forms a viscoelastic network that is responsible for the structure and volume of the bread. Water absorption was the same for landraces (55.3%) and modern varieties (55.4%) and similar to the water absorptions obtained by Schopf and Scherf (2021).

Taken together, these results suggest that there is no difference in the baking quality of both groups, although it must be taken into account that there are various other parameters for determining the baking quality (Geisslitz et al., 2018).

In the PCA (Fig. 5), the landraces EGH, RSL and WEF were located in the negative area of PC1 together with all modern varieties except KWS, which was located on the positive side of PC1 with all the other landraces. This means that these three landraces are similar to the modern varieties in certain characteristics. The proportion of gliadins was lower in EGH (57.6%), WEF (59.1%) and RSL (60.4%) compared to the other landraces (64.0% mean value) and more similar to the mean of the modern varieties (57.6%). The same was true for the proportions of glutenins. The mean glutenin proportions of 23.7%, 21.8% and 21.0% were closer to the mean of modern varieties (22.0%) than the one of landraces (17.4%). Therefore, GLIA/GLUT (2.5, 2.8 and 3.0) were also closer to the mean of modern varieties (2.8) than to the one of landraces (4.3). The mean of HMW-GS was 5.7% for modern varieties and 4.6% for landraces. With 7.1%, 6.3% and 5.9% these values were also closer to the mean of modern varieties. One landrace stood out in particular. The variety EGH had a high bread volume of 708 mL, which is comparable to the one of the modern variety WIW (703 mL). This is particularly interesting when it comes to baking bread, as this variety presumably has similar rheological properties and is therefore easier to process than the other landraces.

Although this study provides new insights into the protein composition and baking quality of common wheat landraces, the comparison to the modern varieties must be handled cautiously. With 14 landraces and only six modern varieties, the statistical evaluation is not ideal. This imbalance means that study findings need to be interpreted critically. The relatively small sample quantity also leads to a greater scattering of results. However, taken together, all studies (including ours) essentially concluded that there were several differences between modern varieties and old varieties in terms of protein composition and baking quality, but that all in all no clear distinction could be made between the two groups.

## 5 Conclusion

The aim of this study was to investigate the protein composition of landraces in comparison to modern varieties to find out, if the breeding process resulted in significant differences. Therefore, 14 landraces and six modern varieties grown in three consecutive years were analyzed. Crude protein content, proportions of ALGL and gluten, water absorption and bread volume did not differ between the two groups. Proportions of gliadins were significantly higher in the group of landraces, whereas those of glutenins were lower. Although GLIA/GLUT was higher and the proportion of HMW-GS were lower in the group of landraces than in the group of modern varieties, the bread volumes of both groups did not differ. The PCA with all data confirmed these results, showing no clear distinction between the two groups. Taken together, no clear distinction could be made between landraces and modern varieties in terms of protein composition and baking quality. This study highlights that common wheat landraces can be a good alternative to modern varieties, especially for organic farming. They offer a large genetic diversity, are regionally adapted and it is possible to manufacture products of equal quality compared to the ones of modern varieties. This is why they offer a great opportunity for small and medium-sized companies in particular to make handmade specialty products. Further, it seems that landraces may not be better tolerated in the context of wheat-related disorders, but further studies focusing on the immunoreactive potential are needed to make a more detailed assessment.

## Funding

The project was supported by funds of the 10.13039/501100005908Federal Ministry of Food and Agriculture (BMEL) based on a decision of the parliament of the Federal Republic of Germany via the Federal Office for Agriculture and Food (BLE) under the Federal Programme for Ecological Farming. Project number 2819OE021 (ReBIOscover).

## CRediT authorship contribution statement

**Nora Jahn:** Conceptualization, Data curation, Formal analysis, Investigation, Methodology, Visualization, Writing – original draft, Writing – review & editing. **Ulla Konradl:** Data curation, Resources, Investigation, Writing – review & editing. **Klaus Fleissner:** Conceptualization, Investigation, Resources, Writing – review & editing. **Sabrina Geisslitz:** Conceptualization, Funding acquisition, Investigation, Project administration, Methodology, Supervision, Writing – review & editing. **Katharina A. Scherf:** Conceptualization, Funding acquisition, Project administration, Resources, Supervision, Writing – review & editing.

## Declaration of competing interest

The authors declare that they have no known competing financial interests or personal relationships that could have appeared to influence the work reported in this paper.

## Data Availability

The processed data required to reproduce the above findings are available within the manuscript and its supplement.
